# Influence of layer separation on the determination of stomach smooth muscle properties

**DOI:** 10.1007/s00424-021-02568-5

**Published:** 2021-04-26

**Authors:** Mischa Borsdorf, Markus Böl, Tobias Siebert

**Affiliations:** 1 Department of Motion and Exercise Science, University of Stuttgart, Stuttgart, Germany; 2 Institute of Mechanics and Adaptronics, Department of Mechanical Engineering, Braunschweig University of Technology, Braunschweig, Germany

**Keywords:** Contractile muscle properties, Uniaxial tensile experiments, Force–velocity relationship, Force–length relationship, Stomach, Separated muscle layer, Organ wall

## Abstract

Uniaxial tensile experiments are a standard method to determine the contractile properties of smooth muscles. Smooth muscle strips from organs of the urogenital and gastrointestinal tract contain multiple muscle layers with different muscle fiber orientations, which are frequently not separated for the experiments. During strip activation, these muscle fibers contract in deviant orientations from the force-measuring axis, affecting the biomechanical characteristics of the tissue strips. This study aimed to investigate the influence of muscle layer separation on the determination of smooth muscle properties. Smooth muscle strips, consisting of longitudinal and circumferential muscle layers (whole-muscle strips [WMS]), and smooth muscle strips, consisting of only the circumferential muscle layer (separated layer strips [SLS]), have been prepared from the fundus of the porcine stomach. Strips were mounted with muscle fibers of the circumferential layer inline with the force-measuring axis of the uniaxial testing setup. The force–length (FLR) and force–velocity relationships (FVR) were determined through a series of isometric and isotonic contractions, respectively. Muscle layer separation revealed no changes in the FLR. However, the SLS exhibited a higher maximal shortening velocity and a lower curvature factor than WMS. During WMS activation, the transversally oriented muscle fibers of the longitudinal layer shortened, resulting in a narrowing of this layer. Expecting volume constancy of muscle tissue, this narrowing leads to a lengthening of the longitudinal layer, which counteracted the shortening of the circumferential layer during isotonic contractions. Consequently, the shortening velocities of the WMS were decreased significantly. This effect was stronger at high shortening velocities.

## Introduction

There are three types of muscles: skeletal, cardiac, and smooth muscles. The latter are predominantly located in the walls of blood vessels and organs of the gastrointestinal, urogenital, and respiratory tract. Smooth muscles are non-striated and cannot be controlled voluntarily. Through complex coordination between contraction and relaxation, they facilitate the propulsion of various contents within the body and regulate the inner pressure of organs. Due to their distinct features and crucial role in the healthy functioning of various vital body systems, research on smooth muscles has been of high physiological and medical interest [1, 53].

Typically, walls of hollow organs consist (from inside to outside) of mucosal, muscular, and serosal layers. A decisive aspect of the study of smooth muscles are active biomechanical properties, including the characteristic force–length relationship (FLR) [15] and the hyperbolic force–velocity relationship (FVR) [20]. Their examination yields experimental data, which are necessary for the development of muscle models [46] and realistic three-dimensional models of organs [23, 42]. These, in turn, can describe the organ in health and disease and advance the knowledge and treatment of various medical conditions.

Active biomechanical properties of muscles are commonly examined through in vitro uniaxial tensile experiments [43, 50], in which muscle forces are measured in one direction only. Typically, muscle tissue is mounted along the muscle direction to a force-measuring device, while muscle activation is initiated through electrical or pharmacological stimulation. Thereby, biomechanical properties of smooth muscles in uniaxial experiments have been examined on tissue specimens, dissected from the bladder [8, 17, 25, 28, 30, 32, 38, 39, 42, 45, 54–58], the stomach [18, 23, 34, 49], the intestine [2, 3, 13, 16, 26, 29, 31], the trachea [7, 35, 47, 48, 51], or from blood vessels [11, 12, 19, 36, 37, 60].

However, the walls of the urinal and gastrointestinal organs contain multiple smooth muscle layers with different muscle fiber orientations [6, 33, 41]. While various studies conducted tests on isolated muscle layers [4, 51, 54], muscle bundles [28, 32, 38, 56], or single smooth muscle cells [18], active biomechanical properties are frequently investigated of strips from which the muscle layers are not separated [8, 16, 30, 34, 49, 58]. In such experiments, narrow strips are cut and mounted so that the examined muscle layer and specifically their muscle fibers are aligned lengthwise to the force-measuring axis of the measuring device. In contrast, the other muscle layers (with deviating muscle fiber directions) are still attached. Therefore, strip activations initiate muscle contractions in more directions than just in the lengthwise axis of the strip. Since the strips are not fixed transversally, this would cause a shortening in that direction. Although generally neglected, a transverse shortening may have a considerable effect on examining biomechanical properties through uniaxial experiments. To date, there has not been a systematic study that investigated the influence of layer separation on the determined muscle properties.

The aim of this study is to examine the influence of muscle layer separation on the determined muscle properties. Therefore, we investigated the active biomechanical properties of smooth muscle strips, consisting of the whole-muscle tissue, and muscle strips, consisting of only one separated muscle layer. Strips were dissected from the ventral fundus of the pig stomach, as this region exhibits two orthogonally oriented, distinct muscle layers [6, 49]. Uniaxial experiments were conducted to investigate muscle properties, including the FLR and FVR.

## Materials and methods

### Experimental setup

**Fig. 1:**
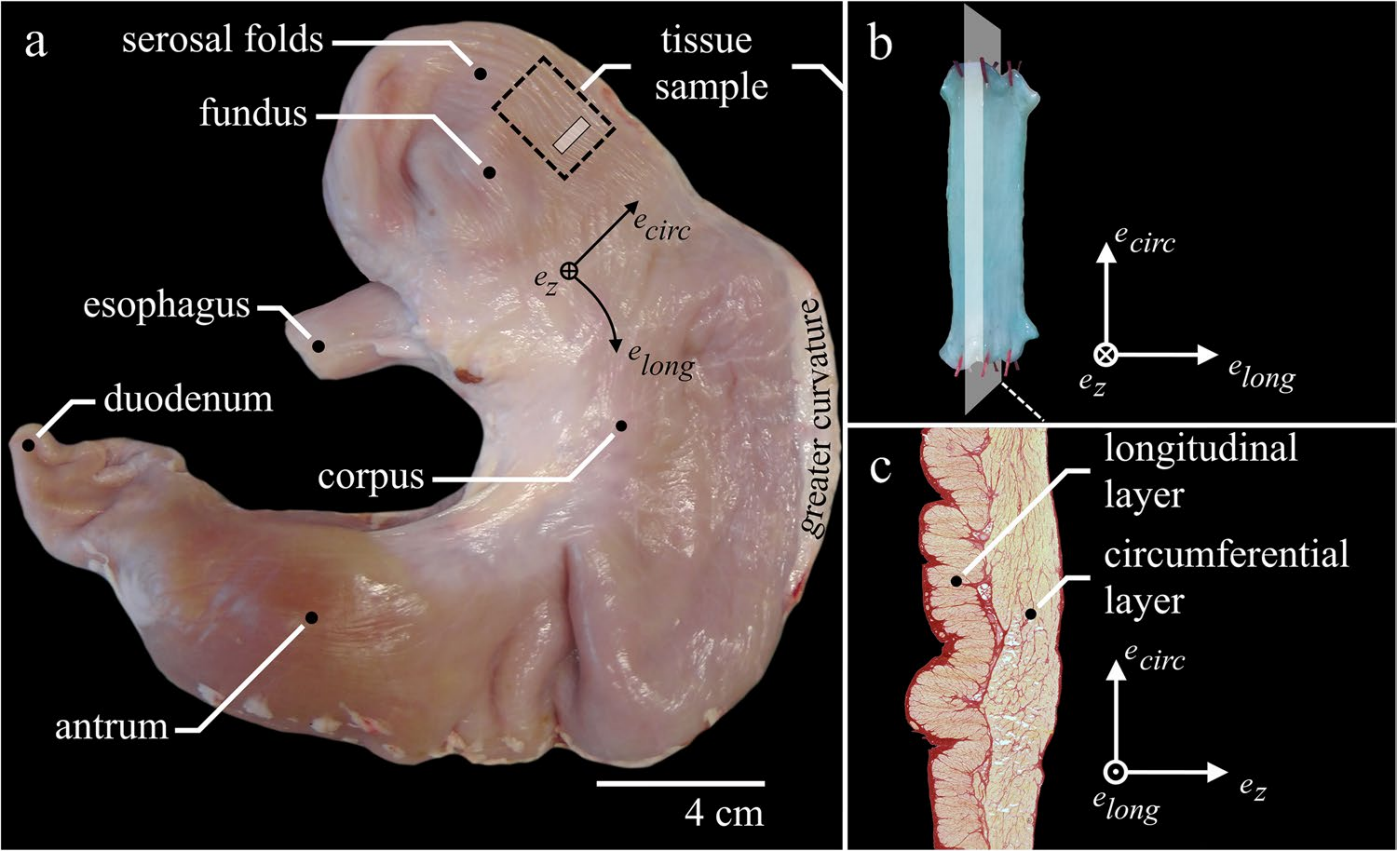
Tissue strip structure and location in the porcine stomach. **a** Ventral view of a porcine stomach with anatomical regions. The dashed black rectangle indicates the location from which tissue strips were dissected. The visible gastric serosal folds run inline with the greater curvature. **b** Tissue strip, mounted in the uniaxial tensile experiments. Note the blue color of the strip was due to the addition of methylene blue to the solution. The transparent white plane illustrates the **c** lengthwise histological cut of a porcine muscle tissue strip from the fundus. The mucosal layer is missing, as it was cut away for the experiments. The thin serosal layer on the outside (left), the outer longitudinal and the inner circumferential muscle layers can be identified

Porcine stomachs of healthy domestic pigs (*Sus scrofa domestica*, age ≈6 month, mass ≈100 kg) were obtained from a slaughterhouse immediately after animal sacrifice. After the death of the animal, stomachs were prepared within 90 min. The experiments were conducted on overall nineteen (n = 19) gastric smooth muscle strips cut from 12 healthy pig stomachs. The experimental setup, handling, and preparation of gastric smooth muscle tissue have been described earlier in detail [23, 49]. An aerated (95% O_2_ and 5% CO_2_) Krebs solution (124 mM NaCl; 5 mM KCl; 2.5 mM CaCl_2_; 15 mM NaHCO_3_; 1.2 mM KH_2_PO_4_, 1.2 mM MgSO_4_, 10 mM C_6_H_12_O_6_; pH 7.3 at 37 °C) was used for storage of the tissue and realization of the experiments at temperatures of 4 °C and 37 °C, respectively. To eliminate spontaneous contractions, the method of adding 50 µM methylene blue to the solution plus illumination was used during experiments [10, 24, 40]. Smooth muscle tissue strips (0.26 ± 0.11 g) of 15 × 7 mm were dissected from the ventral fundus in the circumferential direction (i.e., transversal to the direction of the gastric serosal folds and orthogonal to the greater curvature, see Fig. 1a). Experiments were performed on strips from which only the mucosal layer was removed (whole-muscle strips, WMS), and strips from which the mucosal and the longitudinal muscle layers were removed, leaving only the circumferential smooth muscle layer (separated layer strips, SLS). Both ends of the strip were threaded into small rectangular blocks (10 × 8 × 3 mm) with a braided fishing line. Via these blocks, the strip was mounted vertically to a dual-mode muscle lever system (Aurora Scientific 305C-LR; accuracy: ± 1 mN in force; ± 1 µm in length), to be part of the Aurora Scientific 805A in vitro muscle apparatus. The circumferential layer was aligned along the force-measuring axis e_circ_ (Fig. 1b, c). After the strips were suspended in a relaxed state at a very short length (clearly sagging), they were passively stretched via an isokinetic ramp (5 mm/min) until a passive force of 1–3 mN was reached. After reaching the end of the ramp, the strips were kept at this length, while the passive force development settled to a negligible initial value (< 1 mN). The length of the strips, averaging 10.8 ± 1.3 mm between attachment threads, was determined with a digital sliding caliper (± 0.02 mm accuracy) and was defined as slack length (*L*_*S*_). Muscle contractions were induced by supramaximal electrical stimulations (1000 mA, 100 Hz, 5 ms) for 16 s. The muscle properties were determined after an equilibration period of 30 min [8, 23, 49].

### Determination of muscle properties

Uniaxial experiments were performed in vitro on nineteen (*n* = 19) tissue strips. Table 1 summarizes the number of samples examined for each analyzed parameter. The FLR was investigated by a series of isometric contractions with length increments of 20% *L*_*S*_ until a decrease in force, and hence the descending limb of the FLR, was established. The muscle length at maximum isometric force (*F*_*im*_) was defined as the optimum muscle length (*L*_0_). The ascending and descending limbs of the FLR were fitted using linear regression models (f (x) = mx + b, with x = *L/L*_*S*_) to extrapolate to lengths of zero force generation [36, 60]. The respective distances to *L*_0_ were defined as the width of the ascending (*w*_*asc*_) and descending (*w*_*desc*_) limbs.

**Table 1:** The number of investigated tissue samples of each strip group for the determined muscle properties and parameters. FLR parameters: *L*_0_, *w*_*asc*_, *w*_*desc*_, *p*_*act*_, and *p*_*pass*_. FVR parameters: *v*_*max*_ and *curv*

Stomach Number	Whole-muscle strips		Separated layer strips	
	FLR	FVR	FLR	FVR
1	X		X	X
2			X	X
3	X	X	X	
4	X		X	X
5	X	X		
6	X	X	X	X
7	X	X	X	X
8	X		X	
9			X	X
10	X	X		
11	X	X	X	X
12	X	X		
Number of Samples	10	7	9	7

Before determining the FVR, the strips were stimulated isometrically every 5 min until a stable maximal isometric force generation (deviation < 3% *F*_*im*_) was established. A series of 6 isotonic contractions determined the FVR at *L*_0_ against forces of 0.1–0.9 *F*_*im*_. The data were fitted with Hill’s hyperbolic equation to yield the maximum shortening velocity (*v*_*max*_) and the curvature factor *curv* = *a/F*_*im*_ (*a* describes the force asymptote). Between all contractions, recovery phases of 5 min were interposed, and the “cycling-protocol” by Brenner [9] was applied.

The cross-sectional area (CSA) of the strip at *L*_0_ was determined from the strip volume and the length of the tissue between the stitches. The volume was calculated by its wet weight, assuming a density of 1.05 g/cm^3^ [19]. *F*_*im*_ and the passive isometric force were divided by the strip’s CSA to calculate the maximum active stress (*p*_*act*_) and the corresponding passive stress at *L*_0_ (*p*_*pass*_), respectively.

### Histological observations

Histological analyses were performed exemplarily for one WMS strip to show typical stomach smooth muscle structure. Cross-sectional, lengthwise-sectional, and in-plane slices were stained with Picrosirius Red staining protocol [33], exhibiting two distinct muscle layers. The outer muscle layer contains muscle fibers, oriented in the longitudinal direction, whereas the inner muscle layer consists of muscle fibers in the circumferential direction. The procedure is described in detail in previous studies [6, 30, 49].

### Statistics

For statistical analyses, muscle properties were normalized as follows: (1) Force and length values were divided by individual CSA and *L*_0_, respectively. (2) Velocity data were normalized to optimal muscle length and expressed in *L*_0_*/s*. The strips were differentiated according to their composition (WMS vs. SLS). The biomechanical properties were analyzed using seven parameters (*L*_0_, *w*_*asc*_, *w*_*desc*_, *p*_*act*_, *p*_*pass*_, *v*_*max*_, and *curv*). No indication for deviation from normal distribution was found using the Kolmogorov–Smirnov test with *p* > 0.05. Each parameter was tested for significant differences using Student’s unpaired t-test. A significance level of *p* < 0.05 was used for all analyses. Statistical analyses were carried out using MATLAB (The Mathworks, Inc., Natick, MA, USA).

## Results

**Fig. 2:**
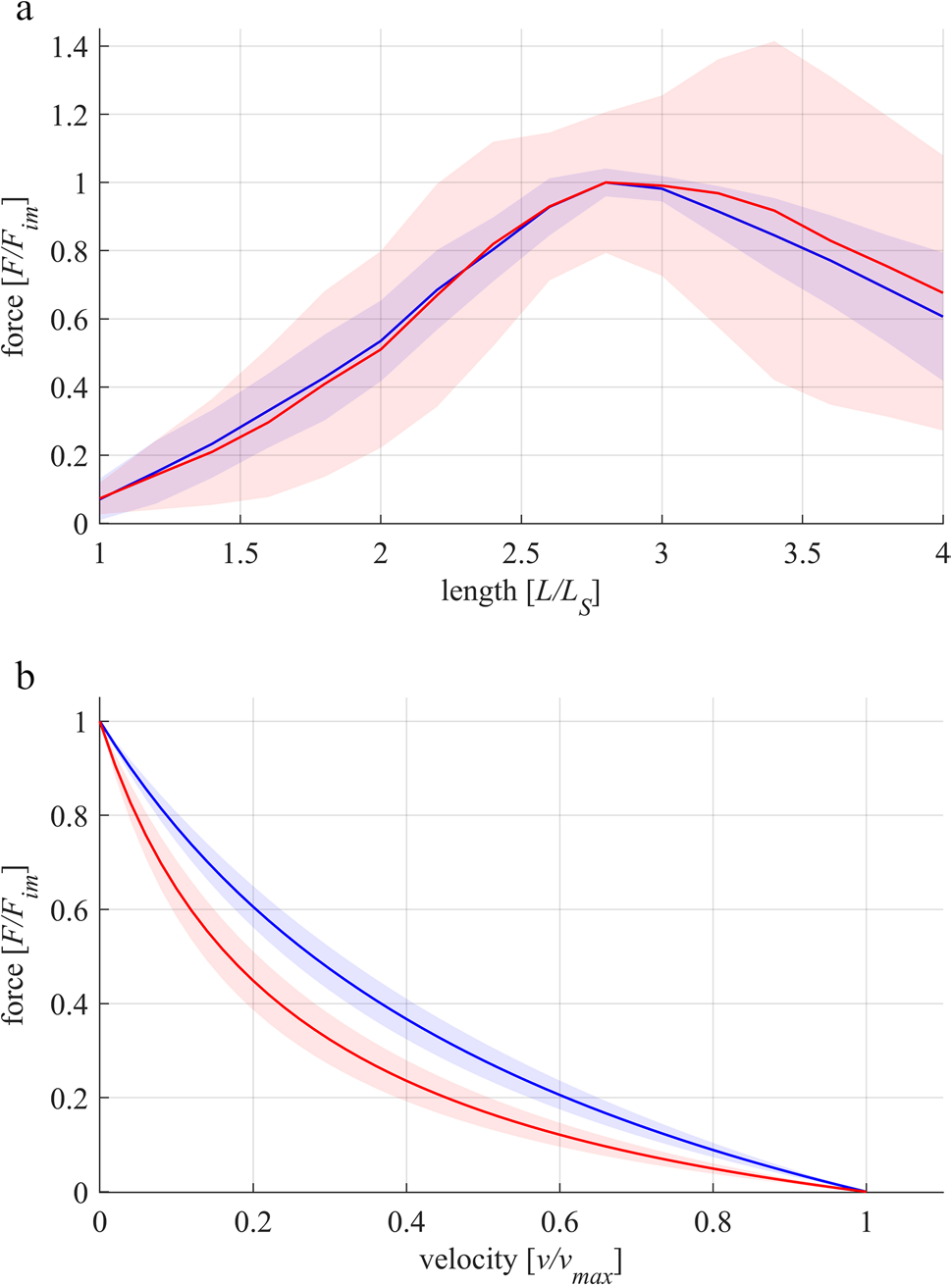
Active muscle properties of whole-muscle strips (WMS, blue color) and isolated layer strips (SLS, red color). The solid curves and light-shaded areas indicate mean values and standard deviations, respectively. **a** Active force–length relationship. Force and length are normalized to maximum isometric force (*F*_*im*_) and slack length (*L*_*S*_), respectively. Isometric force–length measurements comprise *n* = 10 tissue samples for whole-muscle strips and *n* = 9 samples for isolated layer strips. **b** Force–velocity relationship. Velocity and force values are normalized to maximum shortening velocity (*v*_*max*_) and *F*_*im*_, respectively. Based on a series of 6 isotonic contractions, the force–velocity curves of n = 7 tissue samples were fitted using the typical Hill equation [20, 43]

**Fig. 3:**
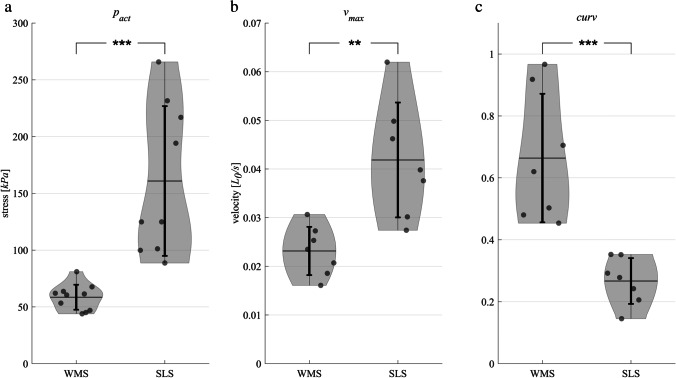
**a** Influence of layer separation on maximum active isometric stress (*p*_*act*_), **b** maximum shortening velocity (*v*_*max*_), and **c** *curv* factor of the force–velocity relationship (FVR). For whole-muscle strips (WMS) and separated layer strips (SLS), mean values ± standard deviations are displayed by the black horizontal line and the vertical error bars, respectively. The transparent black markers indicate the individual data points. The shaded area (violin plot [21]) illustrates the distribution of the data. Brackets and asterisks ( ∗) mark significant differences. Significance levels are marked as ∗  ∗ *p* < 0.01 and ∗  ∗  ∗ *p* < 0.001

The FLR and FVR of whole-muscle strips and separated layer strips are shown in Fig. 2. The shape of the mean active isometric FLR was similar for both groups investigated (Fig. 2a). Specifically, the FLR exhibited a linear ascending limb, a bell-shaped plateau region, and a linear descending limb. No differences have been found for optimum muscle length (WMS: *L*_0_ = 2.86 ± 0.23 *L/L*_*S*_, SLS: *L*_0_ = 2.98 ± 0.43 *L/L*_*S*_). Furthermore, there were no differences in *w*_*asc*_ and *w*_*desc*_ between both groups of strips (Table 2). However, *p*_*act*_ was significantly lower for WMS (58.5 ± 11.5 kPa) compared to SLS (160.9 ± 66.5 kPa) (Fig. 3a).

**Table 2:** Influence of layer separation on optimal length (*L*_0_), the width of ascending (*w*_*asc*_), and descending limb (*w*_*desc*_) of the force–length relationship and passive stress (*p*_*pass*_) at *L*_0_. *n.s.*, not significant

Sample	*L*_0_ (*L/L*_*S*_)	*w*_*asc*_ (*L/L*_0_)	*w*_*desc*_ (*L/L*_0_)	*p*_*pass*_ (kPa)
Whole-muscle strips	2.86 ± 2.32	0.67 ± 0.07	1.29 ± 0.98	20.6 ± 13.6
Separated layer strips	2.97 ± 4.29	0.63 ± 0.05	0.87 ± 0.58	20.3 ± 8.2
*p*	0.461 (n.s.)	0.203 (n.s.)	0.273 (n.s.)	0.944 (n.s.)

**Fig. 4:**
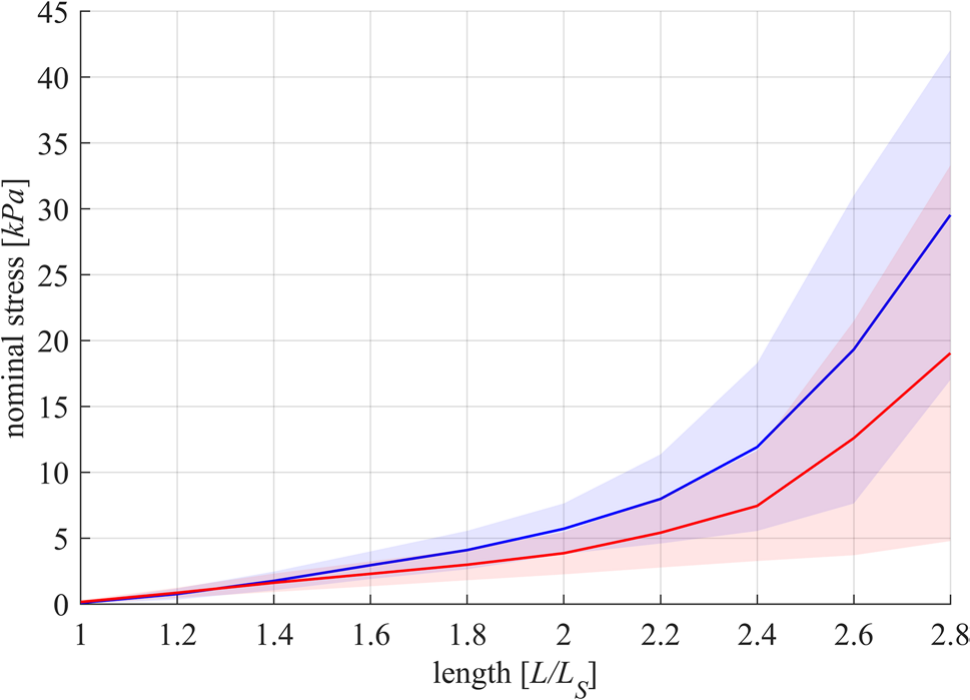
Passive stress-length relationship of whole-muscle strips (blue) and separated layer strips (red). Force is divided by the cross-sectional area (CSA) of the strip at slack length (*L*_*S*_) to obtain nominal stress. Passive stresses are measured right before activation on each length from isometric experiments. The solid curves and light-shaded areas indicate mean values and standard deviations, respectively

The FVR was normalized to *F*_*im*_ and *v*_*max*_ in Fig. 2b. Both groups of strips exhibited a typical hyperbolic shape of the FVR. Maximum shortening velocity was lower (*p* = 0.0025, Fig. 3b) for WMS (*v*_*max*_ = 0.023 ± 0.005 *L*_0_/s) compared to SLS (*v*_*ma x*_ = 0.042 ± 0.012 *L*_0_/s). Furthermore, curvature factor of WMS (*curv* = 0.664 ± 0.21) was significantly higher (*p* < 0.001, Fig. 3c) than that of SLS (*curv* = 0.267 ± 0.076). The passive stress–strain relationships of both groups are displayed in Fig. 4. Both curves were characterized by a course that resembles an exponential increase of stress with muscle length. No statistically significant differences were found between the two groups for *p*_*pass*_.

## Discussion

To our knowledge, there has not been a systematic study that examined the influence of layer separation on smooth muscle strip properties. Therefore, the present study performed in vitro uniaxial tensile experiments on tissue samples from the fundus of the porcine stomach to characterize the FLR and FVR of WMS and SLS. We found apparent differences in muscle properties determined from shortening contractions, such as in the parameters *v*_*max*_ and *curv*. In contrast, there was a negligible influence of layer separation on the normalized FLR parameters determined from isometric contractions. These results are relevant for the determination of realistic muscle parameters, which are required in muscle models. Moreover, the results presented can be used to check which parameters in the existing data are influenced by the interaction of multiple muscle layers.

### Porcine smooth muscle properties—comparison with the literature

The average *p*_*act*_ of 160.9 ± 66.5 kPa generated by separated layer strips can be considered as the pure smooth muscle stress. Histological analyses yielded negligible amount of collagen in the circumferential muscle layer; see Fig. 1c. Observed *p*_*act*_ values are in agreement with stress values smooth muscles typically generate [8, 18, 47, 54]. The FLR displayed the typical bell shape of smooth, skeletal, and cardiac muscles. The extrapolated range, at which the muscle produced forces (0.35 to 2.08 *L*_0_), is similar to examined values of other studies on smooth muscles, which lie between 0.13 and 0.38 *L*_0_ [8, 28, 30, 36, 48, 49, 54, 60] and 1.82 and 2.17 *L*_0_ [8, 30, 36, 44, 49, 60]. The average *v*_*max*_ of 0.023 ± 0.003 *L*_0_/s and 0.042 ± 0.012 *L*_0_/s of WMS and SLS, respectively, are rather low, but within the range of reported values between 0.016 and 0.6 *L*_0_/s of visceral smooth muscles [2, 3, 8, 16, 26–32, 34, 38, 39, 49, 55, 56]. The mean *curv* of 0.664 ± 0.21 and 0.267 ± 0.076 of WMS and SLS, respectively, are in agreement with values of 0.1–0.8 found in smooth [2, 8, 16, 26, 27, 29–31, 34, 37–39, 47–49, 55] and skeletal muscles [43]. The mean *p*_*pass*_ of 20.6 ± 13.6 kPa of WMS at *L*_0_ amount to about 35% of their average *p*_*act*_ (58.5 ± 11.5 kPa), which are in accordance with findings of previous studies on smooth muscles [16, 30, 49].

### Influence of layer separation on smooth muscle properties

**Fig. 5:**
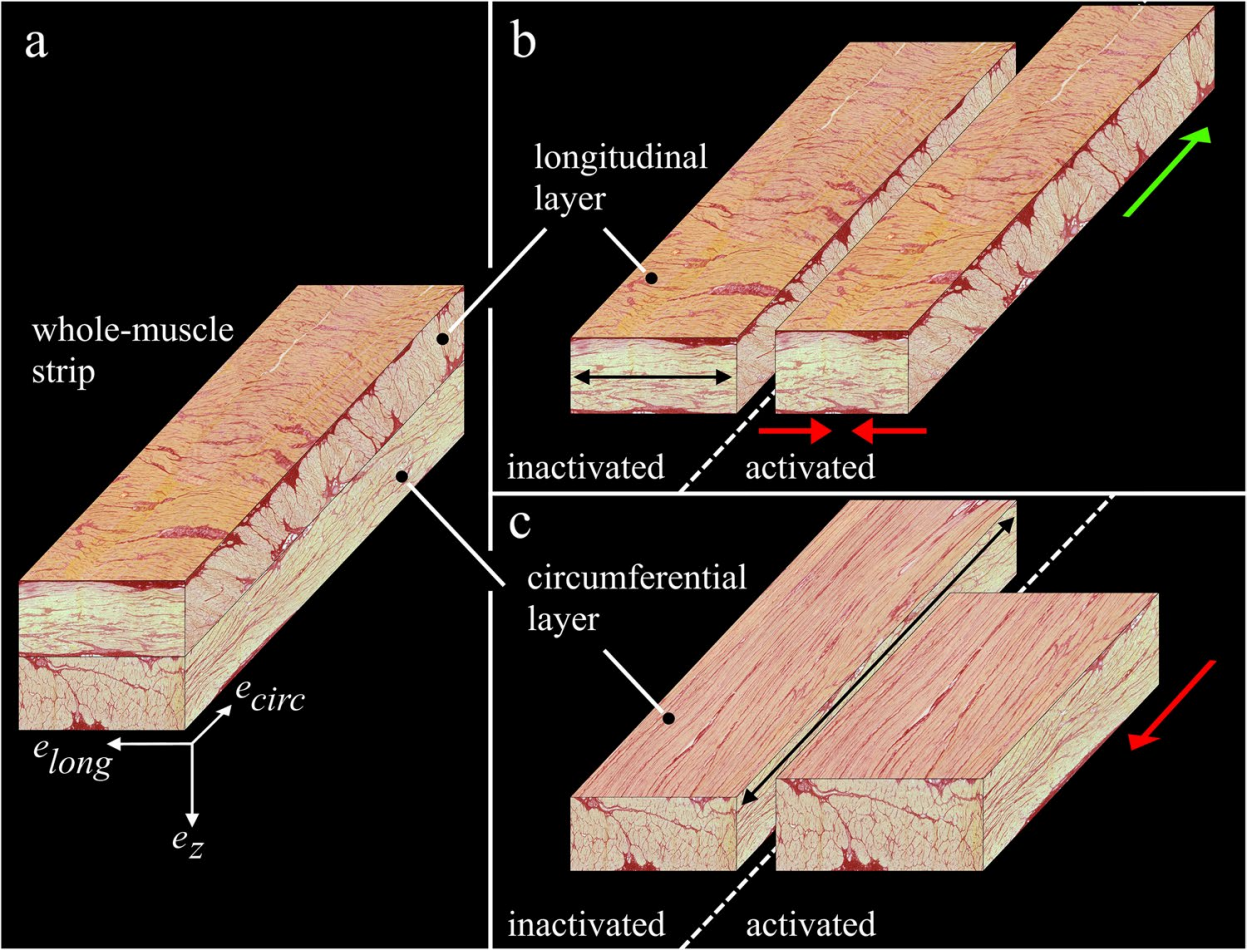
Schematic reconstruction of whole-muscle strips from the porcine stomach during concentric contractions in uniaxial experiments. Strip representation is based on perpendicular histological examinations with Picrosirius Red staining protocol. **a** Whole-muscle strips were cut from the fundus (Fig. 1) lengthwise to the circumferential smooth muscle layer. They consisted of the outer longitudinal layer **b** and the inner circumferential layer **c**. When activated, both layers contracted concentrically along their muscle fiber direction (black arrows in **b** and **c**). While the circumferential layer shortened lengthwise (**c**, red arrow), the longitudinal layer shortened transversally (**b**, red arrows). Due to volume constancy, the narrowing of the longitudinal layer prompted its simultaneous lengthening (green arrow). Through force transmission between the layers, this lengthening counteracted the shortening of the circumferential layer during the concentric contraction. Consequently, the shortening velocity was decreased in WMS

**Fig. 6:**
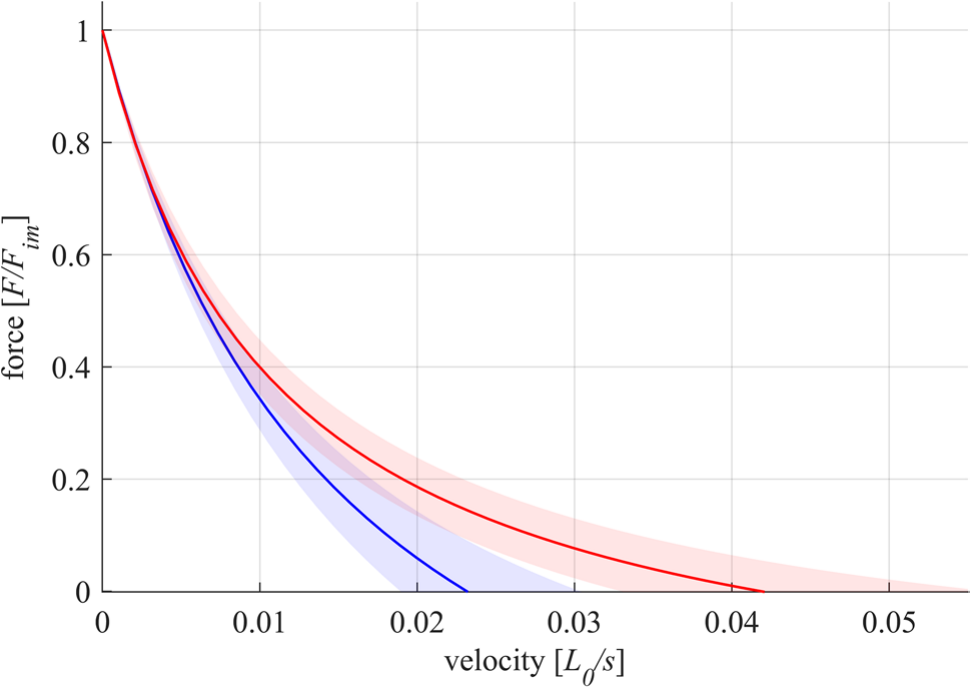
Force–velocity relationship of whole-muscle strips (blue) and separated layer strips (red). Velocity and force values are normalized to optimum muscle length (*L*_0_) and maximum isometric force (*F*_*im*_), respectively. Based on a series of 6 isotonic contractions, the force–velocity curves of n = 7 tissue samples were fitted using the typical Hill equation [20, 43]

The separation of the circumferential smooth muscle layer affected the shortening characteristics of the examined tissue strips, as WMS displayed lower *v*_*max*_ and higher *curv* than SLS (Fig. 3b, c). WMS consisted of the circumferential and the longitudinal muscle layers, which were oriented in the lengthwise (e_circ_) and transversal directions (e_long_) of the strip, respectively (Fig. 5a). The two layers may be regarded as isolated, individual muscle layer strips. The muscle fibers of the longitudinal layer strip run along its narrow side (Fig. 5b, black arrow), whereas the muscle fibers of the circumferential layer strip run along its lengthwise side (Fig. 5c, black arrow). During isotonic contractions, the circumferential layer shortens along the lengthwise axis of the strip (Fig. 5c, red arrow), while simultaneously the longitudinal layer shortens in the transverse direction of the strip (Fig. 5b, red arrows). The transverse shortening of the longitudinal layer causes a narrowing of the strip, which causes the strip to lengthen due to expected volume constancy of the muscle tissue. This lengthening is directed opposite the shortening of the circumferential muscle layer. Similar to myofascial force transmissions in skeletal muscles [22, 61], force transmission occurs between the neighboring smooth muscle layers. The lengthening of the longitudinal layer counteracts the concentric contraction of WMS, reducing its shortening velocity. As SLS does not contain the longitudinal layer, this effect does not apply, and the shortening velocity of SLS is not reduced. Therefore, SLS exhibited higher *v*_*max*_ than WMS (Fig. 3b). The shortening velocity of WMS was reduced more markedly compared to SLS at concentric contractions against low relative forces (high velocities) (Fig. 6).

During isometric contractions (*v* = 0), no or negligible change in WMS strip length is expected. Due to volume constancy, this would result in no or negligible lengthening of the longitudinal layer and thus no or negligible counteracting forces of the longitudinal layer during isometric contractions. Counteracting forces of the longitudinal layer might decrease with decreasing velocity due to velocity-dependent viscoelastic properties of muscle tissue [59] and velocity-dependent damping properties of the FVR.

For the comparison of isometric contractions, the FLR was determined. As a result, the layer separation did not affect the shape of the FLR. However, the *p*_*act*_ was higher in SLS than that in WMS. While both groups consisted predominantly of muscle tissue, the longitudinal layer of the WMS did not generate tensile forces along the measuring force axis during isometric contractions due to the orientation of its muscle fibers in the transversal direction. Therefore, the active stress of WMS was lower due to the higher CSA of the WMS strip. Nevertheless, it remains a question whether the pure muscle stresses of the SLS and the WMS were different if the WMS force will be normalized by the CSA of only the tensile force-generating muscle tissue, i.e., its circumferential layer. However, for a valid analysis of such a parameter, determining CSA of both WMS muscle layers by histological examinations of all tested samples would be necessary, which was not performed in the present study.

The *p*_*pass*_ and the course of the nominal stress with increasing length did not differ between WMS and SLS (Table 2). This suggests an isotropic passive behavior of the smooth muscle tissue in the gastric fundus, agreeing with observations of studies that conducted biaxial experiments on tissue strips from this region [5, 6].

### Relevance for studies on smooth muscle strips

It was shown that the examined muscle properties through uniaxial experiments are dependent on whether the muscle layers of smooth muscle strips are separated or not. From a physiological point of view, it would be preferable to separate the muscle layers, so smooth muscle properties are not influenced by such side effects. However, whole-muscle tissue strips represent more realistic samples for the organ wall in vivo, and hence may be more suitable for the three-dimensional modeling of organs. Furthermore, depending on the organ, or the animal from which the smooth muscle strips are harvested, it may not be possible to separate the muscle layers. For example, some regions of the bladder wall do not contain clearly separate muscle layers but rather multiple, differently oriented muscle fibers [33]. Studies on the stomach of guinea pigs [34] or the intestine of rabbits [13] report difficulties in separating the muscle layers, as this was not possible without potentially damaging the tissue. In this case, biaxial tension testing [14, 33, 52] of muscle tissue is recommended.

In sum, uniaxial experiments are a valid method to determine smooth muscle properties, especially if separated muscle layers were used. However, when conducting experiments on strips with multiple muscle layers, the effect of a transversal shortening needs to be considered and discussed.

## Conclusion

The present study examined the influence of layer separation on the muscle properties of smooth muscle strips from the porcine stomach. SLS displayed higher *v*_*max*_ than WMS, as the contraction of the longitudinal layer of the WMS with its transversally oriented muscle fibers affected the shortening velocity. This effect is weaker with decreasing shortening velocity, which results in a lesser curvature (higher *curv* parameter) of the force–velocity relation of WMS compared to SLS. The FLR showed no differences in shape between the groups. However, SLS generated a higher *p*_*act*_ than WMS. This was due to higher CSA of WMS compared to SLS with the same amount of circumferential muscle layer tissue in WMS and SLS generating force in the direction of the force-measuring axis. The results of this study show that the determination of smooth muscle properties through uniaxial experiments may depend on whether the muscle layers are separated or not.

## Data Availability

The datasets generated during and/or analysed during the current study are available from the corresponding author on reasonable request.

## Abbreviations

CSA: Cross-sectional area
*curv*: Curvature factor
*F*_*im*_: Maximum isometric muscle force
FLR: Force–length relationship
FVR: Force–velocity relationship
*L*_0_: Optimum muscle length associated with *F*_*im*_
*L*_*S*_: Slack length
*p*_*act*_: Maximum active stress at *L*_0_
*p*_*pass*_: Passive stress at *L*_0_
SLS: Separated layer strips
*v*_*max*_: Maximum shortening velocity
*w*_*asc*_: Width of the ascending limb
*w*_*desc*_: Width of the descending limb
WMS: Whole-muscle strips
